# Profiling analysis of circulating microRNA in peripheral blood of patients with class IV lupus nephritis

**DOI:** 10.1371/journal.pone.0187973

**Published:** 2017-11-14

**Authors:** Elkin Navarro-Quiroz, Lisandro Pacheco-Lugo, Roberto Navarro-Quiroz, Hernan Lorenzi, Pierine España-Puccini, Yirys Díaz-Olmos, Lisneth Almendrales, Valeria Olave, Henry Gonzalez-Torres, Anderson Diaz-Perez, Alex Dominguez, Antonio Iglesias, Raul García, Gustavo Aroca-Martinez

**Affiliations:** 1 Grupo de Nefrología, Universidad Simón Bolívar, Barranquilla,Colombia; 2 Centro de Investigación en Salud para el Trópico, Universidad Cooperativa de Colombia, Santa Marta, Colombia; 3 Infectious Diseases Department, J. Craig Venter Institute, Rockville Maryland, United States of America; 4 Unidad de Reumatología, Universidad Nacional de Colombia, Bogotá, Colombia; 5 Clínica de la Costa, Barranquilla, Colombia; Peking University First Hospital, CHINA

## Abstract

Renal involvement in Systemic Lupus Erythematous (SLE) patients is one of the leading causes of morbidity and a significant contributor to mortality. It’s estimated that nearly 50% of SLE individuals develop kidney disease in the first year of the diagnosis. Class IV lupus nephritis (LN-IV) is the class of lupus nephritis most common in Colombian patients with SLE. Altered miRNAs expression levels have been reported in human autoimmune diseases including lupus. Variations in the expression pattern of peripheral blood circulating miRNAs specific for this class of lupus nephritis could be correlated with the pathophysiological status of this group of individuals. The aim of this study was to evaluate the relative abundance of circulating microRNAs in peripheral blood from Colombian patients with LN-IV. Circulating miRNAs in plasma of patients with diagnosis of LN-IV were compared with individuals without renal involvement (LNN group) and healthy individuals (CTL group). Total RNA was extracted from 10 ml of venous blood and subsequently sequenced using Illumina. The sequences were processed and these were analyzed using miRBase and Ensembl databases. Differential gene expression analysis was carried out with edgeR and functional analysis were done with DIANA-miRPath. Analysis was carried out using as variables of selection fold change (≥2 o ≤-2) and false discovery rate (0.05). We identified 24 circulating microRNAs with differential abundance between LN-IV and CTL groups, fourteen of these microRNAs are described for the first time to lupus nephritis (hsa-miR-589-3p, hsa-miR-1260b, hsa-miR-4511, hsa-miR-485-5p, hsa-miR-584-5p, hsa-miR-543, hsa-miR-153-3p, hsa-miR-6087, hsa-miR-3942-5p, hsa-miR-7977, hsa-miR-323b-3p, hsa-miR-4732-3p and hsa-miR-6741-3p). These changes in the abundance of miRNAs could be interpreted as alterations in the miRNAs-mRNA regulatory network in the pathogenesis of LN, preceding the clinical onset of the disease. The findings thus contribute to understanding the disease process and are likely to pave the way towards identifying disease biomarkers for early diagnosis of LN.

## Introduction

Systemic lupus erythematosus (SLE) is a heterogeneous inflammatory chronic autoimmune disorder characterized by progressive involvement of multiple-organ systems with alternating clinical exacerbations and remissions [1]. Though SLE typically cycles through periods of flares and remission, patients often eventually succumb to end-stage kidney or cardiovascular damage [2]. Lupus nephritis (LN) is a common and debilitating feature of SLE. The precise immune mechanisms that drive the progression from benign autoimmunity to LN are largely unknown [3]. The diversity of clinical, serological and immunological symptoms are the result of the activation of varied immune components by complex molecular mechanisms that finely tuned the general process of protein production through DNA transcription and RNA translation; among these, circulating microRNAs play an important role. However, the role of miRNAs in the pathophysiology of lupus nephritis are far from being fully understood [2,4,5].

The prevalence of SLE and the chances of developing lupus nephritis (LN) vary considerably between different regions of the world and different races and ethnicities [6]. Black and hispanic SLE patients have worse outcomes than white patients with SLE, including death and end-stage renal disease (ESRD)[7]. In Colombia, LN-IV is the most prevalent form of lupus nephritis, reaching up to 66% of the total of cases [8]. For many years renal biopsy has remained the gold standard in the first approach to patients with suspected LN. However, renal biopsy is considered an invasive, painful and frightening procedure, and their utility is questionable [9].

There is now increasing interest in exploring epigenetic mechanisms in SLE [5] with notable progress in studies for characterizing the contribution of microRNAs (miRNAs) [10]. miRNAs expression patterns could not only provide a new diagnostic tool as an alternative to renal biopsy, but also the search for contributing circulating miRNAs in the context of renal injury in LN present promising new therapeutic options by targeting miRNAs [11].

One of the major challenges gene therapy applications face clinically is the ability to control the level of expression or silencing of therapeutic genes in order to provide a balance between therapeutic efficacy and nonspecific toxicity due to overexpression of therapeutic protein or microRNAs. Thus, hopefully improved understanding of the implications of microRNAs in the pathogenesis of LN can result in the continued development of more specific and effective treatments [12–14]. In the present study we describe the differences in the abundance of circulating microRNAs among patients with class IV lupus nephritis, patients with lupus without nephritis and healthy individuals.

## Materials and methods

### Samples

The present study was based on a cohort of Colombian patients with lupus nephritis (www.nefrored.org). SLE patients fulfilled at least 4 of 17 SLICC classification criteria, including at least one clinical and one immunologic criterion [15]. Renal histopathology was classified according to the 2003 revised criteria for glomerulonephritis of SLE, which was published by International Society of Nephrology/Renal Pathology Society [16]. Written informed consent was obtained prior to enrolment from all adult individuals participating in the study. Study protocol was reviewed and approved by the ethics review board at Simon Bolivar University.

### Sample processing

Whole blood (10 mL) from subjects was collected via a direct venous puncture into tubes with ethylenediaminetetraacetic acid (EDTA) as an anticoagulant. Blood was processed for the isolation of plasma within 4 h of collection. Samples were processed by spinning at 2,000 x g for 10 min at room temperature. Then, the plasma was transferred to a RNase-free tube and stored at –80°C.

### RNA isolation from plasma

Plasma samples were thawed on ice. Total RNA was isolated from 600 μl of plasma using miRVana PARIS Kit (Ambion) following the manufacturer instructions. RNA concentration was measured by Nanodrop 2000 spectrophotometer (Thermo Scientific) and stored at −80°C. All materials and solutions were handled in RNase-free conditions. All solutions were prepared in RNase-free water and all methods were carried out in accordance with the approved guidelines.

### miRNAs sequencing and differential gene expression analysis

Illumina barcoded miRNA sequencing libraries were prepared from extracted miRNA samples using TruSeq Small RNA Library Prep Kit (Illumina®) following manufacturer instructions. Sequencing libraries were sequenced in two pools of 15 samples each per sequencing runs with a NextSeq apparatus to generate ~16 million single-end 75 bp reads per sample. Afterwards, sequencing reads were processed with CLC Genomics Workbench software (https://www.qiagenbioinformatics.com/products/clc-genomics-workbench/) to obtain the final counts of miRNAs present in teach sample. Briefly, adapter sequences were removed from sequencing reads and the remaining sequences were compared against the miRBase database (www.miRBase.org) and the Ensembl human non-coding RNA annotation (version GRCh37.75) with CLC for miRNA gene identification, annotation and quantification. Differential gene expression analysis between groups of interest was carried out with the R package EdgeR [17]. Micro RNAs showing at least a two-fold change in their relative abundance with a Benjamini-Hochberg corrected p-value (FDR) ≤ 0.05 were considered significant [18].

### Identification of miRNAs potentially implicated in the regulation of signaling pathways in patients with lupus nephritis IV

For the identification of the networks and pathways of the selected miRNAs we used DIANA miRPath (v3.0) [19]. We evaluated miRNA dysregulation in LN using miR2Disease [20], PhenomiR [21] for information about miRNAs exhibiting a differential regulation in disease and other biological processes and HMDD [22], for information of experimentally supported human miRNA-disease association data from genetics, epigenetics, circulating miRNAs and miRNA-target interactions.

### Transcription factor target analysis

Validated TF-miRNA interactions and their regulation (activation or repression) were exported from TransmiR database [23]. On the other hand, the predicted interactions of TF-miRNA and TF-gene were determined by retrieving the promoter sequences from all the miRNAs and genes previously identified. The promoter region was defined as a 2 kbp sequence starting 1.5 kbp upstream from the transcription start site (TSS) and terminating 0.5 kbp downstream of the TSS. TSS miRNA was obtained using miRStart [24].

### Regulatory network construction

In the miRNA-based network, we included miRNAs, their targets, TFs regulating these miRNAs and their targets as well as the type of interaction between these molecules. We assumed that all miRNAs repress their targets, unless otherwise indicated in TransmiR [23] miRNA target activation is possible but remains a rare event. We also assumed that TFs activate their targets, unless otherwise indicated in TransmiR. The gene-based network was created in a similar fashion. The networks were constructed and visualized using Cytoscape (version 3.2.0) [25].

## Results

### Clinical characteristics of patients

A total of 21 individuals were enrolled in this study: four patients withLN-IV, ten patients with SLE without nephritis (LNN group) and seven individuals without autoimmune disease (including LES) (Controls) (Table 1).

**Table 1:** Clinical and laboratory characteristics in each group study.

CHARACTERISTICS	LN-IV	LNN	CONTROLS
Gender	F (100)%	M (10%) F(90)%	M (14.2.%) F(85.8)%
Mean age in Years (Range)	36 (13–89)	35.72 (15–25)	30.72 (15–25)
Mean proteinuric flares 24 hr	1573.29 mg	413,61 mg	134,84 mg
Antinuclear antibodies	N (15%) P (85%)	P(100%)	0%
Cilindruria	Hialine (2.5%). Negative (85%). Positive (5%) Hematic (10%). Granulose (5%)	N/A	N/A
Creatinine	1.5484(1.07)	N/A	N/A
Anti-ds DNA	N (47.5%); P (52.5%)	N (6.25%); P (93,75%)	N/A
SLEDAI	≥8	>5	N/A

M: Male; F: Female; N: Negative; P: Positive; N/A: Not apply; Mean.

### Differences in the relative abundance of circulating miRNAs in individuals with LN class IV, LNN and controls

We found 24 miRNAs differentially expressed when we compared the LN-IV group with the individuals of the control group, 16 miRNAs were found overexpressed and 8 miRNAs showed reduced levels between these two groups. 8 of these miRNAs have been previously associated with the regulation of SLE (hsa-miR-361-3p [26], hsa-miR-145-5p [27], hsa-miR-410-3p [28], hsa-miR-125 [29], hsa-miR-199a-5p [26], hsa-miR-550b-2-5p [5], hsa-miR-106a-5p [30], and hsa-miR-183-5p [31]). The circulating miRNA with the highest relative abundance was miR-153-3p (LogFC of 6.6857) and the miRNA with the lowest relative expression was miR-6741-3p (LogFC of -4.7567) (Table 2)

**Table 2:** Comparison of circulating miRNA abundance between LN class IV and controls subjects.

ID	logFC^&^	p-value	FDR*
miR-183-5p	-3.4469	1.4E-06	0.0008
miR-145-5p	3.1853	3.1E-06	0.0009
miR-584-5p	2.9965	6.0E-06	0.0011
miR-6087	6.0385	2.6E-05	0.0036
miR-1260b	3.1143	6.8E-05	0.0076
miR-375-3p	-2.4741	1.7E-04	0.0115
miR-550b-2-5p	-2.3703	1.9E-04	0.0118
miR-199a-5p	2.1479	3.7E-04	0.0186
miR-6741-3p	-4.7567	4.9E-04	0.0207
miR-4511	-4.1905	5.1E-04	0.0207
miR-4732-3p	-2.0623	7.7E-04	0.0237
miR-410-3p	2.3532	9.4E-04	0.0268
miR-485-5p	4.7818	9.6E-04	0.0268
miR-543	4.5887	1.1E-03	0.0286
miR-125b-5p	2.2824	1.2E-03	0.0296
miR-550a-5p	-2.9970	1.3E-03	0.0303
miR-153-3p	6.6857	1.3E-03	0.0305
miR-323b-3p	2.8748	1.5E-03	0.0342
miR-106a-5p	-2.6934	1.7E-03	0.0360
miR-7977	3.0530	1.9E-03	0.0381
miR-369-5p	3.1559	2.0E-03	0.0390
miR-589-3p	3.9175	2.4E-03	0.0436
miR-3942-5p	4.2715	2.6E-03	0.0458
miR-107-3p	2.9574	2.9E-03	0.0472

^&^ logarithm of fold-change

*False Discovery Rate

Comparative analysis of the relative abundance of plasma circulating miRNAs between the LN-IV group and LNN group we identified two miRNAs that showed significantly different abundances, miR-375-3p (LogFC of 2.34) and miR-758-3p (LogFC -3.75) (Table 3). In addition, A similar analysis between SLE patients without nephritis and controls found two additional miRNAs that were differentially represented in plasma samples, miR-4433b-5p and miR-550a-5p with LogFC of 2.86 and -2.72, respectively, when we compared the LNN group and the control group values (Table 4).

**Table 3:** Comparative analysis of circulating miRNA between LN class IV and LNN patients.

ID	logFC^&^	p-value	FDR*
miR-375-3p	2.34	0.000199	0.046
miR-758-3p	-3.75	0.00024	0.046

^&^ logarithm of fold-change

*False Discovery Rate

**Table 4:** Comparison of circulating miRNAs between LNN patients and control individuals.

ID	logFC^&^	p-value	FDR*
miR-4433b-5p	2.86	0.00023	0.031
miR-550a-5p	-2.72	0.00028	0.032

^&^ logarithm of fold-change

*False Discovery Rate

### MicroRNA targets

Next, we determined, validated and predicted protein-coding gene targets of identified miRNAs with different relative abundance using miRTarBase [32] and TarBase [19] databases containing experimentally validated miRNA-target interactions. We found that miR-106a-5p presented higher numbers of genes that have been experimentally associated with a total of 1,199 genes (S1 Table). In contrast, eight miRNAs (hsa-miR-153-3p, hsa-miR-6087, hsa-miR-3942-5p, hsa-miR-7977, hsa-miR-323b-3p, hsa-miR-410-3p, hsa-miR-4732-3p and hsa-miR-6741-3p) had no experimentally validated targets described to date.

**Fig 1:**
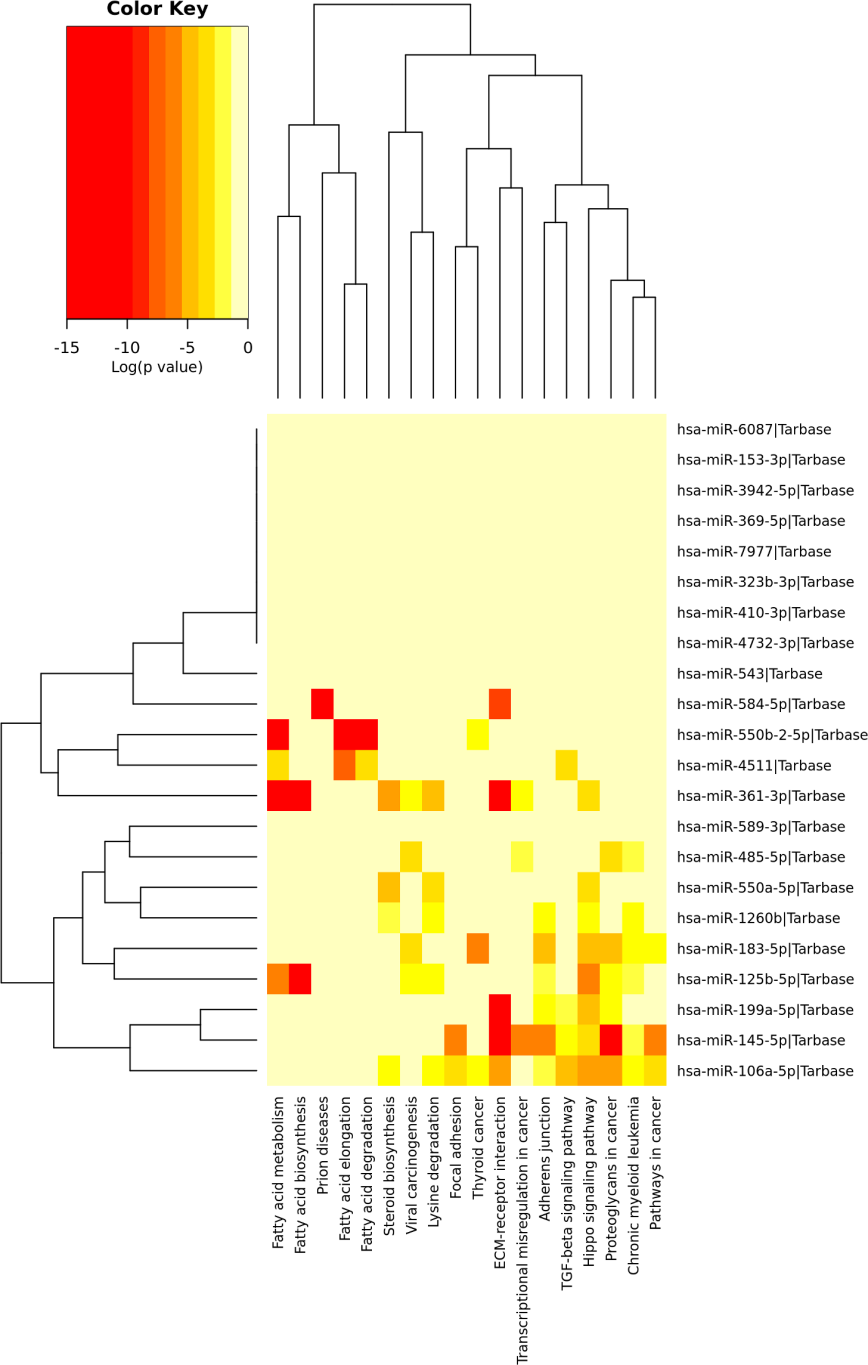
Heat map KEGG pathway with gene regulated by miRNAs with significant differential relative abundance between LN-IV patients and controls.

We used The KEGG to construct a pathway enrichment of experimentally validated miRNA target genes with relative abundance between LN-IV and the control group. Many metabolism networks were found to be involved, including pathways differentially represented LN-IV: fatty acid metabolism, fatty acid biosynthesis, fatty acid elongation, fatty acid degradations,steroid synthesis, ECM-receptor interaction, focal adhesion, adhesion binding, etc (Fig 1).

### Construction of the LN-associated miRNA-based network

**Fig 2:**
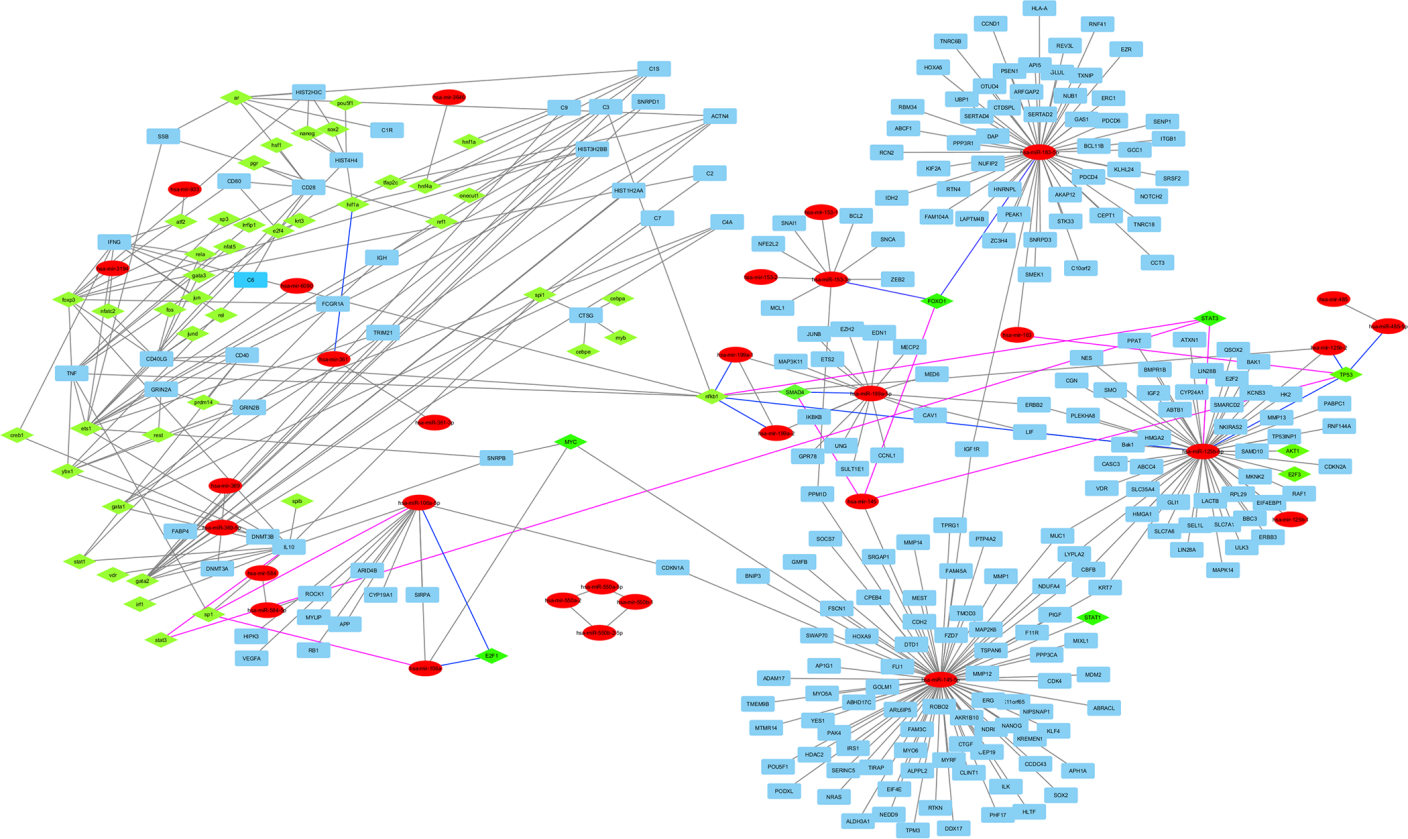
Network interactions between miRNAs, targeted regulatory genes and transcription factors in patients with LN-IV. The red ellipse nodes are miRNAs, the blue rectangle nodes are miRNA´s gene targets, and the green rhombus nodes are transcription factors. Gray line shows regulation of miRNAs on targeted genes by direct miRNA-mRNA interaction, the purple lines represent activation of transcription of miRNAs by transcription factors and blue lines represent down-regulation of transcriptional factors by miRNA-TF interaction.

A network was assembled based on interactions between miRNAs, targeted regulatory genes and transcription factors, which contains 352 nodes (48 miRNAs, 259 targeted genes and 45 transcription factors) and 379 direct interactions (232 direct interactions between miRNAs-targeted genes, 119 interactions between transcription factors–genes and 27 miRNA-miRNA interactions). In total, network analysis indicated that miRNAs were part of a complex regulatory system in LN-IV. For example, miR-125b-5p regulates 49 targeted genes and interacts with 3 TF and miR-145-5p regulates 63 genes and interacts with 5 TF (Fig 2).

## Discussion

In the present study, we describe differences in the relative abundance of circulating miRNAs in plasma among patients with class IV lupus nephritis (LN-IV), patients with lupus without nephritis (LNN), and healthy individuals (CTL). We found significant differences in the abundance of 24 miRNAs when comparing LN-IV patients with control subjects, two miRNAs when comparing LN-IV with LNN, and 16 miRNAs when comparing LNN and controls. Alterations in the relative abundance of miR-589-3p, miR-1260b, miR-4511, miR-485-5p, miR-584-5p, miR-543, miR-153-3p, miR-6087, miR-3942-5p, miR-7977, miR-323b-3p, miR-4732-3p and miR-6741-3p, and its possible association with lupus nephritis is described for the first time.

Likewise, miR-107-3p was found 5.9 (logFC = 2.95) times more abundant in patients with LN-IV than in healthy individuals. This miRNA has experimentally supported interactions with 2,187 genes, the majority of these expressed in kidney and CD20 positive B lymphocytes. Also interacts with “ZEB2”, which are DNA-binding transcriptional repressors of the E-cadherin (Cdh1) gene [33]. Downregulation of E-cadherin decreases E-cadherin-mediated cell–cell adhesion and promotes the migration of epithelial kidney cells. [34]

This miRNA also represses the expression of VPS4A by direct interaction with its mRNA [35], which mediates scission of microvesicles from the T-cell plasma membrane, events that mediate adaptive cellular immunity and regulate antibody responses dependent on intercellular contacts between T cells and antigen-presenting cells (APCs), then B cells bearing cognate MHC class I peptide complexes (pMHC) receive T cell receptors(TCRs) from T cells and initiate intracellular signals in response to isolated synaptic microvesicles [36]. We believe that the altered abundance of miR-107-3p in plasma of patients with LN-IV could contribute to the pathogenesis of this disease by a combined effect, which begins with the disruption of local cell-cell interaction in the kidney epithelium by the deficiency of E-cadherin. This leads to invasion and infiltration of the surrounding tissue, reaching and penetrating the interior of blood or lymphatic vessels (intravasation), with the consequent transport of these cells from the epithelium of the kidney through the bloodstream, which could lead to an over stimulation of the T cells with defective TCR sorting.

The miRNA miR-375-3p was found 5.54 (log FC = -2.77) times less in patients with LN-IV than in healthy individuals, and 4.68 (log FC = 2.34) times more in patients with LN-IV than LNN. This miRNA have experimentally supported interactions with 290 genes, the majority of these expressed in kidney and lymph nodes, and interacts with “CTCF”. This protein is known to bind insulators and exhibits an enhancer-blocking and barrier function, and more recently, it also contributes to the three-dimensional organization of the genome. CTCF can also serve as a barrier against the spread of DNA methylation and histone repressive marks over promoter regions of tumor suppressor genes [37]. There are CpG islands in the upstream region of the miR-375 gene. DNA hypermethylation is observed in the CpG island of ERα-positive breast cancer cells showing high expression of miR-375, whereas DNA hypomethylation and histone H3 K9 dimethylation are observed in the CpG islands of ERα-negative breast cancer cells. CTCF binds to unmethylated DNA in the CpG islands of ERα-negative cells and induces silencing of miR-375 expression [38] similar to what we found in patients with LN-IV and LNN. The expression of SLK, a Ste20-related kinase, is regulated by miR-375 [39], so low abundance of this miRNA could produce overexpression of SLK able to induce stress fiber disassembly, dysregulate cytoskeletal organization in responses to apoptotic stimuli in patients with LN-IV and LNN. That dysregulation of the cytoskeleton in podocytes of LN-IV patients would be represents a common pathway in the pathogenesis of proteinuria.

We observed that hsa-miR-153-3p was 13.36 (log FC = 6.68)-fold more abundant in patients with LN class IV than in controls and therefore, through its interaction with the transcription factor fox01, it could be decreasing the expression of hsa-miR-183-5p, which was found to be 6.88 (log FC = 3.44) times less abundant in patients with class LN-IV than in controls, and promoting in the same way an increase in the abundance of miR-145-5p, which we found 6.36(log FC = 3.18) times more in patients with class LN-IV than in controls.

Lupus nephritis is more likely caused by the effects of several miRNAs rather than a single miRNA and therefore, it is useful to infer the miRNAs synergistic effects and investigate gene regulation mechanisms at a system-wide level in the miRNA–miRNA synergistic networks.

Equally, we highlight the presence of an E2F1–miRNA feedback loop mechanism, which can be associated with the pathogenesis of SLE, within which it is the negative feedback mechanism of miR-106a (which showed a low relative abundance log FC = -2.69) on E2F1. E2F1 regulates the expression levels of five miRNAs belonging to two genomic miRNA clusters that are very similar to each other (miR-17 ~ 92 and miR-106a ~ 363) [40]. This cluster have on average experimentally supported interactions with 1,500 genes, the majority of these expressed in kidney, lymph nodes, B cells and T cells and interacts with TF E2F1, EGR1, MYC, MYCN, SP1 and ERS1. E2F1 has previously been described as a potential therapeutic target for systemic lupus erythematous[41]. The miRNA miR-106a inhibits the expression of transforming growth factor (TGF)-beta receptor 2 [42]. TGF has multiple suppressive actions on T cells, B cells, macrophages, and other cells, and increased levels correlate with protection and/or recovery from autoimmune diseases [43].

At the same time, miR-199a-1, miR-199-2, mir-125b and miR-125b-5P are negatively regulated by the transcription factor NFKB1, which regulates genes associated with the pathogenesis of SLE such as TNF, IFNG, C3 and CD40LG [44]. Furthermore, In this study we found that miRs-106a-5p was downregulated [45] and miRs-145-5p was upregulated with a log FC of 3.19 [46]. These miRNAs are associated with the regulation of the gene transcription factor STAT3, which has been observed overexpressed during hyperactivity of T cells of patients with LES. STAT3 plays a central role in differentiating follicular T helper cells and Th17, two subsets that orchestrate autoimmune responses in SLE. [47].

Among the miRNAs associated with positive regulation of the EEF2 gene that showed a significant change in their abundance in plasma samples of patients with lupus nephritis class IV were: hsa-miR-584-5p [48], hsa-miR-589-3p [49], hsa-miR-145-5p [48], hsa-miR-183-5p [48], Hsa-miR-125b-5p [35] and hsa-miR-106a-5p [50].

In conclusion, we identified 24 novel differentially abundant miRNAs in the plasma of patients with class IV lupus nephritis (hsa-miR-589-3p, hsa-miR-1260b, hsa-miR-4511, hsa-miR-485-5p, hsa-miR-584-5p, hsa-153-3p, hsa-miR-6087, hsa-miR-3942-5p, hsa-miR-7977, hsa-miR-323b-3p, hsa-miR-4732-3p, hsa-miR-6741-3p) that are good candidates for further assessment as diagnostic biomarkers of class IV lupus nephritis. Additionally, based on previous experimental validations of miRNA-miRNA and miRNA-Transcription Factor interactions, there are many possible implications in the pathophysiology of lupus nephritis, resulting from these alterations in miRNA levels. These results contribute to the understanding of this complex idiopathic disease.

Our results show the existence of a characteristic miRNA signature in plasma from patients with LN class IV that could potentially be used as a molecular tool for the diagnosis of this class of nephritis.

## Supporting information

S1 Table Numbers of genes that have been shown to be regulated bymiRNAs in patients with Class IV lupus nephritis. (DOCX)
