# Supplementary material for: Profiling analysis of circulating microRNA in peripheral blood of patients with class IV lupus nephritis
Source: PLoS One. 2017 Nov 14;12(11):e0187973. doi: 10.1371/journal.pone.0187973 (PMC5685598; doi:10.1371/journal.pone.0187973)
Supplement: S1 Table — (DOCX) [file pone.0187973.s001.docx]

S1 Table. Numbers of genes that have been shown to be regulated by miRNAs in patients with Class IV Lupus Nephritis.

| ID | Genes |
| --- | --- |
| hsa-miR-106a-5p | 1199 |
| hsa-miR-125b-5p | 1150 |
| hsa-miR-183-5p | 913 |
| hsa-miR-145-5p | 511 |
| hsa-miR-361-3p | 508 |
| hsa-miR-589-3p | 485 |
| hsa-miR-1260b | 456 |
| hsa-miR-199a-5p | 342 |
| hsa-miR-4511 | 284 |
| hsa-miR-550a-5p | 247 |
| hsa-miR-485-5p | 165 |
| hsa-miR-584-5p | 127 |
| hsa-miR-543 | 82 |
| hsa-miR-550b-2-5p | 26 |
| hsa-miR-369-5p | 6 |
| hsa-miR-153-3p | 0 |
| hsa-miR-6087 | 0 |
| hsa-miR-3942-5p | 0 |
| hsa-miR-7977 | 0 |
| hsa-miR-323b-3p | 0 |
| hsa-miR-410-3p | 0 |
| hsa-miR-4732-3p | 0 |
| hsa-miR-6741-3p | 0 |
